# Central and peripheral analgesic active components of triterpenoid saponins from *Stauntonia chinensis* and their action mechanism

**DOI:** 10.3389/fphar.2023.1275041

**Published:** 2023-10-16

**Authors:** Ji-Hong Gong, Chang-Ming Zhang, Bo Wu, Zi-Xun Zhang, Zhong-Yan Zhou, Jia-Hui Zhu, Han Liu, Yi Rong, Qian Yin, Ya-Ting Chen, Rong Zheng, Guang-Zhong Yang, Xiao-Fei Yang, Su Chen

**Affiliations:** ^1^ Key Laboratory of Cognitive Science of State Ethnic Affairs Commission, Hubei Key Laboratory of Medical Information Analysis and Tumor Diagnosis and Treatment, College of Biomedical Engineering, South-Central Minzu University, Wuhan, China; ^2^ Gynecology Department, Hubei Maternal and Child Health Hospital, Wuhan, China; ^3^ College of Pharmacy, South-Central Minzu University, Wuhan, China

**Keywords:** stauntonia chinensis, triterpenoid saponins, analgesia, neural signal transduction, transient receptor potential vanilloid 1

## Abstract

Triterpenoid saponins from *Stauntonia chinensis* have been proven to be a potential candidate for inflammatory pain relief. Our pharmacological studies confirmed that the analgesic role of triterpenoid saponins from *S. chinensis* occurred via a particular increase in the inhibitory synaptic response in the cortex at resting state and the modulation of the capsaicin receptor. However, its analgesic active components and whether its analgesic mechanism are limited to this are not clear. In order to further determine its active components and analgesic mechanism, we used the patch clamp technique to screen the chemical components that can increase inhibitory synaptic response and antagonize transient receptor potential vanilloid 1, and then used *in vivo* animal experiments to evaluate the analgesic effect of the selected chemical components. Finally, we used the patch clamp technique and molecular biology technology to study the analgesic mechanism of the selected chemical components. The results showed that triterpenoid saponins from *S. chinensis* could enhance the inhibitory synaptic effect and antagonize the transient receptor potential vanilloid 1 through different chemical components, and produce central and peripheral analgesic effects. The above results fully reflect that “traditional Chinese medicine has multi-component, multi-target, and multi-channel synergistic regulation”.

## 1 Introduction

*Stauntonia chinensis*, a plant belonging to the family Lardizabalaceae, is a traditional Chinese medicine, known as “Ye Mu Gua” (Feng et al., 2019). Its roots, stems, and leaves have anti-nociceptive and anti-inflammatory effects that have been evaluated *in vivo* (Gao et al., 2009). At present, the point injection of *S. chinensis* has the most significant clinical analgesic effect and is used to relieve cancer pain (Yan, 2017), various neuropathic pain (Gao and Yuan, 2019), limb pain (Ma et al., 2012), and rheumatic pain (Gou and Li, 2016).

Although the main raw material for this injection is *Stauntonia chinensis*, the specific effective components of this injection are unclear. Since 1984, adverse reactions to *S. chinensis* injections have been reported in succession. After the application of *S. chinensis* injection, patients had allergic reactions, such as eosinophilia, shortness of breath, and palpitations. Moreover, Ye Wenbo et al. have reported that at a concentration of >500 mg/L, *S. chinensis* injections were harmful to the cells (Zhang et al., 2010). Therefore, exploring the active ingredients with a clear analgesic mechanism from the raw material of *S. chinensis* is crucial for the development of safer and more effective analgesic drugs.

A previous investigation of *S. chinensis* has demonstrated that this evergreen herb is rich in triterpenoid saponins (Wang et al., 1989a; Wang et al., 1989b; Wang et al., 1990; Wang et al., 1991). Gao et al. and Zhang et al. confirmed the anticancer, anti-inflammatory, and analgesic effects of the triterpenoid saponins extracted from *S. chinensis* (TSS) (Zhang et al., 1998; Gao et al., 2008). We also confirmed that TSS specifically impairs the threshold of thermal- and chemical-stimulated acute pain. We analyzed its analgesic mechanism and observed that it selectively increased spontaneous inhibitory synaptic release and gamma-aminobutyric acid (GABA)-induced charge transfer in mouse cortical neurons (Chen S., et al., 2018). Additionally, we discovered that its peripheral analgesic effect was closely related to the modulation of the capsaicin receptor, transient receptor potential vanilloid l (TRPV1) (Chen et al., 2022).

To determine the analgesic active components and their action mechanism of TSS, we first extracted and separated a variety of triterpenoid saponin components from TSS and then screened the components that could increase miniature inhibitory postsynaptic current (mIPSC) in cortical neurons and modulate TRPV1 in dorsal root ganglion (DRG) neurons by conducting patch clamp experiments. Subsequently, we established the animal models of pain, and determined the analgesic effects of the screened components using *in vivo* animal experiments. Finally, we further determined the analgesic mechanism of the screened components through patch clamp and molecular biology experiments. Our results indicated that the components C10 and C9 from TSS exhibited analgesic effects. C10 showed analgesic effect by increasing the mIPSC frequency that related to GABA_A_ receptors, whereas C9 eased the pain by modulating TRPV1 in DRG neurons. These results suggest that TSS exerts corresponding analgesic effects through different components.

## 2 Materials and methods

### 2.1 Selected TSS components

In our previous study, a variety of triterpenoid saponin components from *S. chinensis* was obtained and identified by using the settled extracted and separated methods (Chen et al., 2014). In this paper, eight known triterpene glycoside and two new compounds were extracted, separated, and identified. In the early stages, we specifically studied the analgesic effects of compounds 6 and 7 separately. Unfortunately, these two compounds did not have analgesic effects, and did not show the modulatory effect on mIPSC and TRPV1 in the patch clamp pre-experiments, so they were not included in the scope of screening compounds in this paper.

The remaining eight components were 28-*O*-β-D-glucopyranosyl-(1→6)-β-D- glucopyranosyl-30-norhederagenin (Compound **1**), 3-*O*-β-D-xylopyranosyl-(1→3)-α-L- rhamnopyranosyl-(1→2)-α-L-arabinopyranosyl-30-norhederagenin (Compound **2**), 3-*O*-α- Larabinopyranosyl-30-norhederagenin (Compound **3**), sinofoside A (Compound **4**), yemuoside YM11 (Compound **5**),3-*O*-β-D-xylopyranosyl-(1→3)-α-L-rhamnopyranosyl-(1→2)-α-L-arabinopyranosyl hederagenin (Compound **8**), hederasaponin D (Compound **9**), and 28-*O*-β-D-glucopyranosyl-(16)-β- D-glucopyranosyl-hederagenin (Compound **10**). In order to further explore the analgesic mechanism of TSS, we screen the effective analgesic ingredients from the monomeric components of TSS in the following experiment. The extraction, separation, and identification of the above components were completed by Professor Guang-zhong Yang. The structural identification, extraction process, and purity of the components are detailed in the references (Chen et al., 2014).

### 2.2 Experimental animals

The Kunming mice and Sprague–Dawley (SD) rats used in this study were purchased from the Hubei Research Center of Laboratory Animals [Grade SPF, SCXK (Hubei) 2015-0018]. For behavioral experiments, animals acclimatized to the laboratory conditions for at least 1 week prior to experimentation. The mice and rats were housed at a temperature of 22°C ± 2°C under a 12 h light/12 h dark cycle and maintained with food and water. All experimental procedures involving mice and rats were performed under a protocol approved by the animal research ethics committee of South-Central Minzu University (No 2021-scuec-013).

### 2.3 Cell culture

#### 2.3.1 Cortical neurons

Cortical neurons were dissociated from newborn pups of Kunming mice as described previously (Wang X., et al., 2020). Briefly, the mouse cortical neurons were dissected and digested with 0.25% trypsin-EDTA (Ethylene Diamine Tetraacetic Acid) (Gibco) at 37°C for 12 min, then plated on glass coverslips coated with poly-L-lysine (Sigma) at a density of 80,000 cells per 12-mm × 12-mm. Cells were cultured at 37°C with 5% CO2 in MEM (Minimum Essential Medium) with the addition of 2% (v/v) B27 (Gibco), 0.5% (w/v) glucose (Sigma), 100 mg/L transferrin (Sigma), 5% (v/v) fetal bovine serum (Gibco), and 2 mM Ara-C (Sigma). Electrophysiological analysis was performed after the neurons were cultured for 13, 14 days.

#### 2.3.2 DRG neurons

DRG neurons were dissociated from SD rats as described previously (Chen et al., 2022). One-month-old SD rats (80–120 g) were stunned by heavy blow on the head and decapitated. L4-L6 lumbar DRGs were dissected and digested with Trypsin (0.3 mg/mL)/Collagenase I (1 mg/mL) at 37°C for 25–30 min. Neurons were mechanically dissociated with gentle pipetting. DRG neurons were plated on poly-D-lysine-coated coverslips and maintained in DMEM (dulbecco’s modified eagle medium) supplied with 10% fetal bovine serum (Gibco), 3.7 g/L NaHCO_3_, and 1% penicillin/streptomycin for 2 h before patch-clamp recordings.

### 2.4 Electrophysiological recordings

#### 2.4.1 Recordings in cortical neurons

A HEKA EPC10 amplifier (HEKA) was used for electrophysiological recordings in whole-cell patch clamping mode as described previously (Wang X., et al., 2020). Patch pipettes were generated from borosilicate glass capillary tubes (World Precision Instruments, Inc.) by using a P-97 pipette puller (Sutter). Whole-cell pipette solution was prepared by 120 mM CsCl, 10 mM HEPES (2-[4-(2-hydroxyethyl)piperazin-1-yl] ethanesulfonic acid), 0.3 mM Na-GTP (guanosine triphosphate), 10 mM EGTA (glycol-bis-(2-aminoethylether)-N,N,N′,N′-tetraacetic acid), and 3 mM Mg-ATP, adjusted pH to 7.2 with CsOH; the cell bath solution contained 140 mM NaCl, 5 mM KCl, 2 mM MgCl_2_, 2 mM CaCl_2_, 10 mM glucose, and 10 mM HEPES-NaOH, adjusted pH to 7.4 with NaOH. The osmotic pressure of the pipette and cell bath solutions were adjusted to 305 and 315, respectively. To isolate inhibitory postsynaptic currents (IPSCs), 20 µM 6-cyano-7-nitroquinoxaline-2,3-dione (CNQX) and 50 µM AP-50 were supplied to the bath solution. To isolate the miniature excitatory postsynaptic current (mEPSC), 100 µM picrotoxin (PTX) was supplied to the bath solution. To monitor mIPSC and mEPSC, 1 µM tetrodotoxin (TTX) was added to the bath solution, blocking action potential. Evoked IPSCs were recorded with 90 µA stimulus pulses. During the recording, different concentrations of drugs were used to incubate neurons according to the needs of the different experimental requirements in the text. GABA-evoked release was measured with 200 µM GABA applied to the neurons for 20 s as previous reported (Chen S., et al., 2018). Current signals were low-pass filtered at 2 kHz (Bessel) and digitized at 10 kHz (Bessel).

#### 2.4.2 Recordings of capsaicin-induced TRPV1 currents in DRG neurons

TRPV1 currents were recorded by using an EPC10 amplifier (HEKA). DRG neurons were placed in an open recording bath filled with bathing solution contained 145 mM NaCl, 5 mM KCI, 2 mM CaCl_2_, 1 mM MgCl_2_, 10 mM HEPES, and 10 mM D-glucose, and pH was adjusted to 7.4. The osmolarity was adjusted to 315–325 mOsmol/L. Glass electrodes (recording electrodes, 1.65 ± 0.05 μm outer diameter) were pulled with a P97 puller (Sutter) and filled with an internal solution contained 140 mM KCl, 2 mM MgCl_2_, 10 mM Na_2_ATP, 2.5 mM CaCI_2_, 10 mM EGTA, and 10 mM HEPES, and pH was adjusted to 7.2. The osmolarity was adjusted to 305–315 mOsmol/L. All testing was performed at approximately 22 °C.

The clamp voltage (Vh) was set to - 60 mV for all experiments, and capsaicin (CAP)-evoked currents were recorded in voltage clamp mode. Capsaicin-evoked whole-cell currents were filtered and the signal sampling frequency was 1 kHz. Membrane potentials were expressed as absolute values (millivolts, mV), and TRPV1 currents were expressed as absolute values (nanoamperes, nA) or multiples of changes compared with basal values. Capsaicin was dissolved into stock solution of 1 mmol/L by dehydrated alcohol, and then diluted into 1 μmol/L by the external solution. Capsazepine (CPZ), the competitive antagonist of the TRPV1 receptor, was prepared at a concentration of 10 μmol/L using the same method. All drugs were administered through a multi-channel rapid micro drug delivery system (Yibo Life Science Instrument).

### 2.5 Animal behavior

#### 2.5.1 Hot plate test

In brief, we used a hot/cold plate stimulator (UGO BASILE S.R.L Biological Research Apparatus 2036 Gemonio VA Italy). The temperature of the hot plate was maintained at 55°C ± 0.5°C during the test. One day before the test, we selected the mice with a pain threshold of 5–30 s. The negative control group was injected with isotonic saline and the positive control group was injected with aspirin (20 mg/kg intraperitoneally). C10 (20 μM) and C9 (4 μM) were injected respectively in the experimental group with a content of 0.2 mL/10 g. Before the drug administration, the pain threshold of each mouse was measured twice and its mean value was taken as the basic threshold. The pain threshold of mice in each group was measured 30, 60, 90, and 120 min after drug administration. If there was no licking reaction in mice within 60 s, it was recorded as 60 s.

#### 2.5.2 Acetic acid-induced writhing test

Acetic acid was used to induce pain of peripheral origin. The negative control group was injected with isotonic saline and the positive control group was injected with TSS (20 mg/kg intraperitoneally). C10 (20 μM) and C9 (4 μM) were injected respectively in the experimental group with the content of 0.2 mL/10 g. The mice were pretreated with the above different drugs 30 min before acetic acid injection. The mice were placed in a glass cylinder and the number of writhing responses in mice for 15 min was recorded as the pain threshold after intraperitoneal injection of 1% acetic acid.

#### 2.5.3 Formalin and capsaicin tests

The method for the formalin test was same as before (Chen S., et al., 2018). The formalin test was made by subcutaneous injection of 5% formalin (20 μL/paw) into the right posterior leg. The nociceptive response produced by mice after capsaicin injection was mainly licking the injection site, so the time spent licking the paw was recorded as the pain threshold. The negative control group was injected with isotonic saline and the positive control group was injected with TSS (20 mg/kg intraperitoneally). C10 (20 μM) and C9 (4 μM) were injected respectively in the experimental group with a content of 0.2 mL/10 g. The mice were pretreated with the above different drugs 30 min before formalin injection. Formalin injection can cause two stages of pain sensation, the first stage lasting for about 5 min immediately after injection, and the second stage lasting for about 15–45 min after injection. The mice were placed in a glass cylinder and the licking times of mice in the two stages were recorded separately.

The capsaicin test was made by subcutaneous injection of 200 μM capsaicin (20 μL/paw) into the right posterior leg. The pain threshold was the time of licking paw after capsaicin injection. The negative control group was injected with isotonic saline and the positive control group was injected with TSS (20 mg/kg intraperitoneally). C10 (20 μM) and C9 (4 μM) were injected respectively in the experimental group with a content of 0.2 mL/10 g. The mice were pretreated with the above different drugs 30 min before capsaicin injection. The mice were placed in a glass cylinder and the licking time of mice within 5 min from the injection of capsaicin was recorded.

#### 2.5.4 Complete freund’s adjuvant (CFA)-induced inflammatory pain model

Adult male SD rats (180–200 g) were placed in a glass coverslip of 56 × 16 × 30 cm for 30 min to adapt to the environment before testing pain thresholds. The thermal paw withdrawal latency was measured by use of a full-automatic plantar heat stimulation (KW-600, Nanjing Calvin). Each rat was measured three times with an interval of 5 min. The mean value of the three measurements was defined as the thermosensitive threshold. Rats with a threshold of less than 5 s were eliminated. If there was no licking reaction in mice within 30s, it was recorded as 30s.

500 μL Complete Freund’s adjuvant (CFA) and 500 μL isotonic saline were pumped and mixed in the syringe until they became a milky white and sticky liquid. The skin of the right hind paw of the rats was wiped with a cotton ball moistened with 75% ethanol. Then, the CFA model was made by subcutaneous injection of configured CFA (20 μL/paw) into the right hind paw. After 48 h CFA injection, we tested the thermal paw withdrawal latency to observe if the pain model was successfully made. The negative control group was injected with isotonic saline and the positive control group was injected with aspirin (20 mg/kg intraperitoneally). C10 (20 μM) and C9 (4 μM) were injected respectively in the experimental group with a content of 0.2 mL/10 g. On the first day of administration, the thermosensitive threshold was measured 30, 60, 90 and 120 min after drug administration to determine the optimal drug onset time. All the drugs were continuously administered for 5 days.

### 2.6 Immunofluorescence and western blot

After the CFA-induced inflammatory pain model behavioral test on the fifth day, the adult male SD rats were decapitated. Half of the DRGs were post-fixed with 4% paraformaldehyde. The DRGs were embedded using paraffin, and then sliced with a thickness of 7 µm. The prepared tissue slices were dewaxed, dehydrated, and antigen repaired. Then, the slices were cleaned with TBS (pH 7.4Tris-HCl buffer) and incubated with 10% donkey serum (antGene ANT051) at room temperature for 20 min. We used rabbit anti TRPV1 antibody (Abcam, 1:500 with QuickBlock™ Primary Antibody Dilution Buffer for Immunol Staining, Beyotime) as the primary antibody. We used Alexa Fluor^®^594 donkey anti-rabbit lgG (H + L) (Thermo Fisher A21207, 1:400 with QuickBlock™ blocking buffer for Immunol staining, Beyotime) as the secondary antibody. Then, the sections were washed with TBS again and DAPI (4′,6-diamidino-2-phenylindole) was added (Roche 216,276, 1:500). After washing, the slices were sealed and photographed under a fluorescence microscope (Olympus, DP72, Japan). Five random visual field images with a magnification of 400 times for each sample were selected for measurement. The mean optical density (MOD) of each visual field was calculated by ImageJ software.

The other half of the DRGs were also removed and stored at −80°C. DRG tissues were dissociated by strong RIPA Lysis Buffer (50 mM Tris-HCl pH 8.0.150 mM NaCl, 1% TritonX-100,1% PMSF). The DRG tissues were ground on ice and centrifuged at 12,000 rpm for 10 min at 4°C. The supernatant was removed and the protein concentration of DRG tissues measured using the BCA (bicinchoninic acid) protein assay kit. The protein supernatant was added with 5 × protein loading buffer and boiled at 100°C for 10 min in a metal bath. The protein samples were loaded with 10 μg total protein per lane and separated on 10% sodium dodecyl sulfate-polyacrylamide gelelectrophoresis and transferred to 0.45 μm PVDF (polyvinylidene fluoride) membranes (Merck KGaA, Darmstadt, Germany). At room temperature, the membranes were blocked with TBST (pH 7.4 Tris-HCl buffer with the addition of Tween) containing 5% skim milk for 1 h. The membranes were washed three times for 5 min with TBST. We used rabbit polyclonal antibody to TRPV1 (1:500 in 5% skimmed milk powder/TBST, Absin, abs147070) as the primary antibody and GAPDH (glyceraldehyde-3-phosphate dehydrogenase) Mouse McAb (1:1000 in 5% skimmed milk powder/TBST, proteintech, 60,004-1-lg) as the internal control antibody. We used goat anti-rabbit IgG HRP (1:10,000 in 5% skimmed milk powder/TBST, biosharp, BL003A) as the secondary antibody to TRPV1. We used goat anti-mouse IgG HRP (1:10,000 in 5% skimmed milk powder/TBST, biosharp, BL001A) as the secondary antibody to GAPDH. A gel imaging analysis system (Bio-Rad, United States) was used to capture the signals. Image processing and analysis software in Java was used for analysis of gray scale values.

### 2.7 ELISA

The DIV13 cortical neurons were used for ELISA (enzyme linked immunosorbent assay) test. Firstly, the culture medium of neurons were removed. Neurons were washed with PBS (phosphate buffer solution), three times, maintained with 1 μM C10 medium in 37°C for 1 h, washed with PBS again, and then shattered by ultrasound within new 1 mL PBS, destroying cells and releasing cAMP (Cyclic adenosine monophosphate). After centrifugation at 4°C, the supernatant was carefully collected. The cAMP levels of neurons treated with or without C10 were tested using the mouse cAMP ELISA kit (ZC-38246). The experiment was repeated three times independently.

Three groups of rats were used for the experiments; one group was a normal control group, one group was used to prepare CFA-induced inflammatory pain models as described above, and the other group was continuously administered C10 (20 μM, 0.2 mL/10 g) for analgesia based on the CFA model. After continuous treatment for 5 days, the cerebral cortex was obtained from rats and the GABA content in each group’s tissues was measured using an GABA ELISA kit (ZC-37903).

### 2.8 Statistical analysis

Data were expressed as the mean ± standard error of the mean (SEM) and analyzed though SPSS software (version 17.0) and Prism 6.01 (GraphPad). Comparison of data between two groups was performed using *t*-tests. Comparison between multiple sets of data was performed using analysis of variance (ANOVA) and Dunnett’s test. Statistical significance was determined as *p* < 0.05. All specific statistical methods and results are presented in the figure legends.

## 3 Results

### 3.1 Selected TSS components capable of modulating mIPSC and TRPV1

#### 3.1.1 Selected TSS components capable of increasing mIPSC in cortical neurons

**FIGURE 1:**
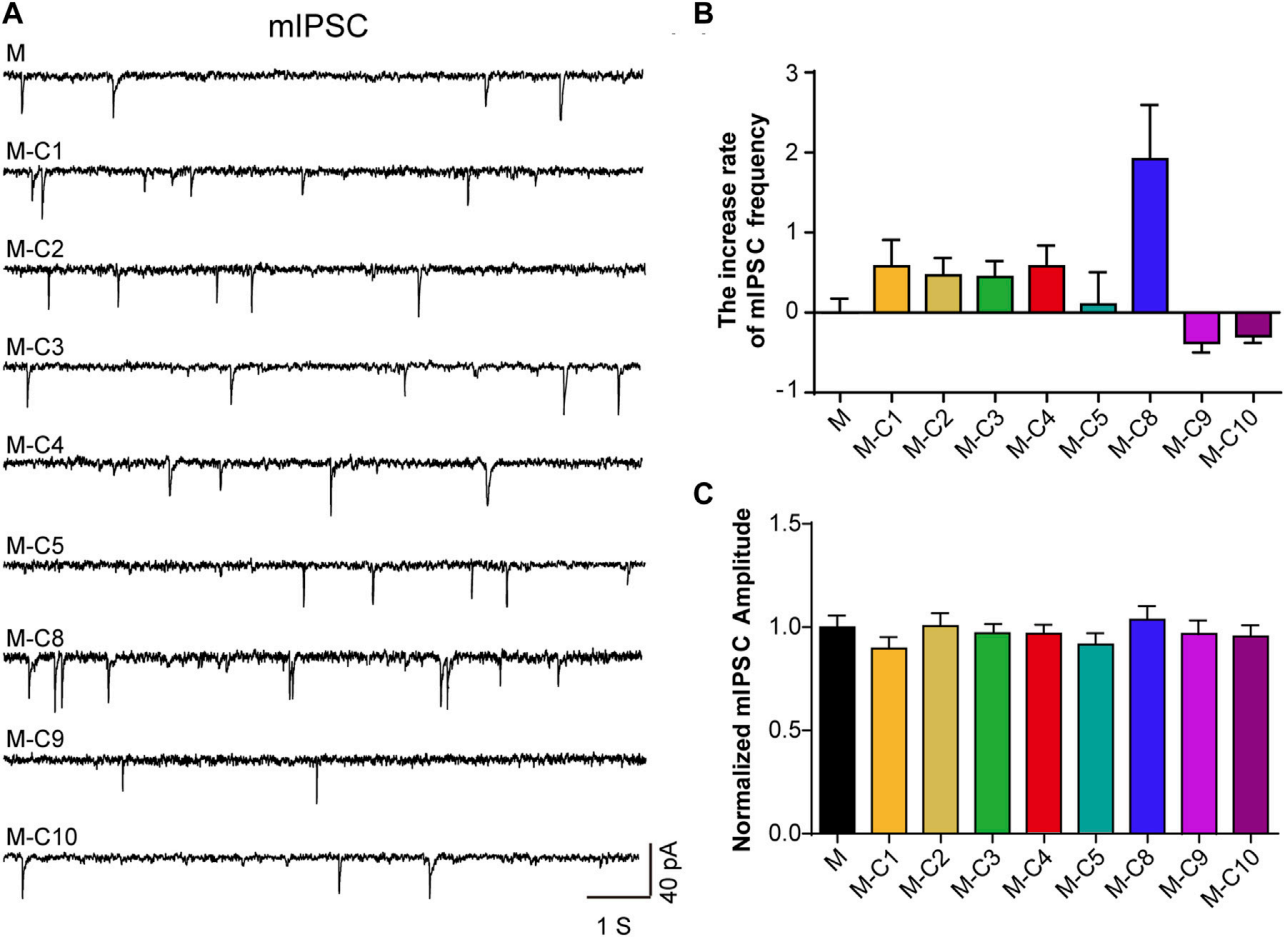
C10 and C9 are the candidates of the effective monomers for TSS increasing mIPSC. **(A)** Example traces of mIPSC were recorded from the cultured cortical neurons that were incubated with seven monomers (2 µM) for 60 min, independently. M represents the mixture of eight monomers (each with a concentration of 2 µM); the combination of C and the number represents a certain compound; M-C number means the mixture of eight monomers with a certain compound removed. **(B, C)** Statistical summary of the increase rate of mIPSC frequency and normalized amplitude of mIPSC described in **(A)**. At least 21 cells of each group were analyzed from six or more independent cultures. Statistical assessments were performed by one-way ANOVA and Dunnett’s test comparing each group to the M group.

Pain sensation is closely related to signal transduction of neurons in the cerebral cortex. Our previous studies have revealed that the analgesic effect of TSS is likely due to an enhancement of mIPSC in mouse cortical neurons (Chen S., et al., 2018). In order to identify the ingredients with analgesic effects, we used a reverse screening method, removing one component from the mixed eight component solution each time and then testing the effects of the remaining seven component mixtures on neurons using a patch-clamp. We found that the increased frequency of mIPSC was reversed after the absence of C10 and C9 components, respectively (Figures 1A–C), suggesting C10 and C9 as the potential analgesic active ingredients in TSS.

#### 3.1.2 Selected TSS components capable of antagonizing TRPV1 in DRG neurons

**FIGURE 2:**
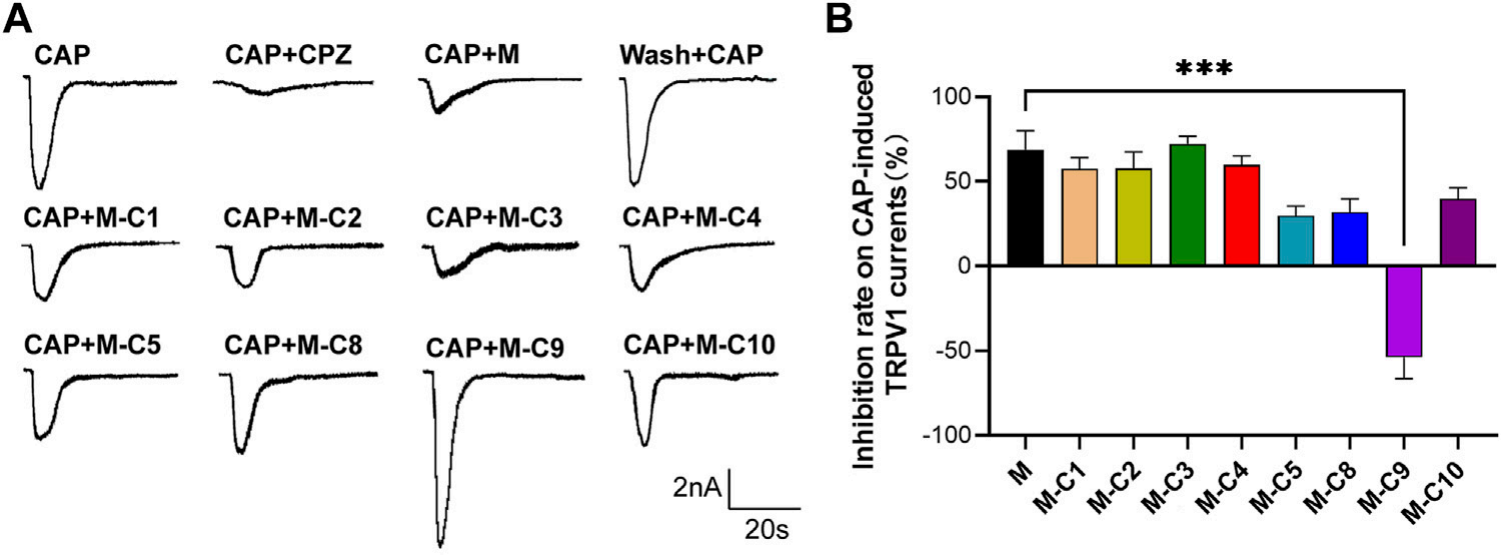
C9 is the candidate of the effective monomers for TSS inhibiting the TRPV1 currents. **(A)** Example traces of TRPV1 currents induced by CAP were recorded from the rat DRG neurons that were treated with random seven monomers (2 µM), independently. M represents the mixture of eight monomers (each with a concentration of 2 µM); the combination of C and the number represents a certain compound; M-C number means the mixture of eight monomers with a certain compound removed. **(B)** Statistical summary of normalized CAP-induced TRPV1 currents. At least six neurons of each group were analyzed from at least 10 rats. Statistical assessments were performed by one-way ANOVA and Dunnett’s test comparing each group to the M group; ***, *p* < 0.001.

Studies have demonstrated that TSS made from *S. chinensis* has an analgesic effect, and that its analgesic mechanism may be related to the modulation of TRPV1 (Chen et al., 2022). To further explore the pharmacological components that inhibit CAP-induced TRPV1 currents in DRG neurons, the above-mentioned reverse screening method was used. The effects of eight saponin component mixtures and seven other saponin component mixtures on CAP-induced TRPV1 currents were tested (Figures 2A, B). The recorded current was almost completely suppressed by capsazepine (CPZ), proving that it was the CAP-induced TRPV1 current. The results of the patch-clamp experiments also showed that the CAP-induced TRPV1 current was reduced by applying the eight saponin component mixtures, which was consistent with the effects of TSS. Interestingly, the inhibitory effect was abolished with the absence of C9 component, indicating that C9 was likely the key component of the TSS that acted on TRPV1.

### 3.2 Analgesic effects of C9 and C10 on different pain models

To determine whether the C10 and C9 components have analgesic effects, the hot plate test, acetic acid-induced writhing test, formalin, capsaicin, and complete Freund’s adjuvant (CFA)-induced chronic inflammatory tests were conducted in mice as follows.

#### 3.2.1 Hot plate test

**FIGURE 3:**
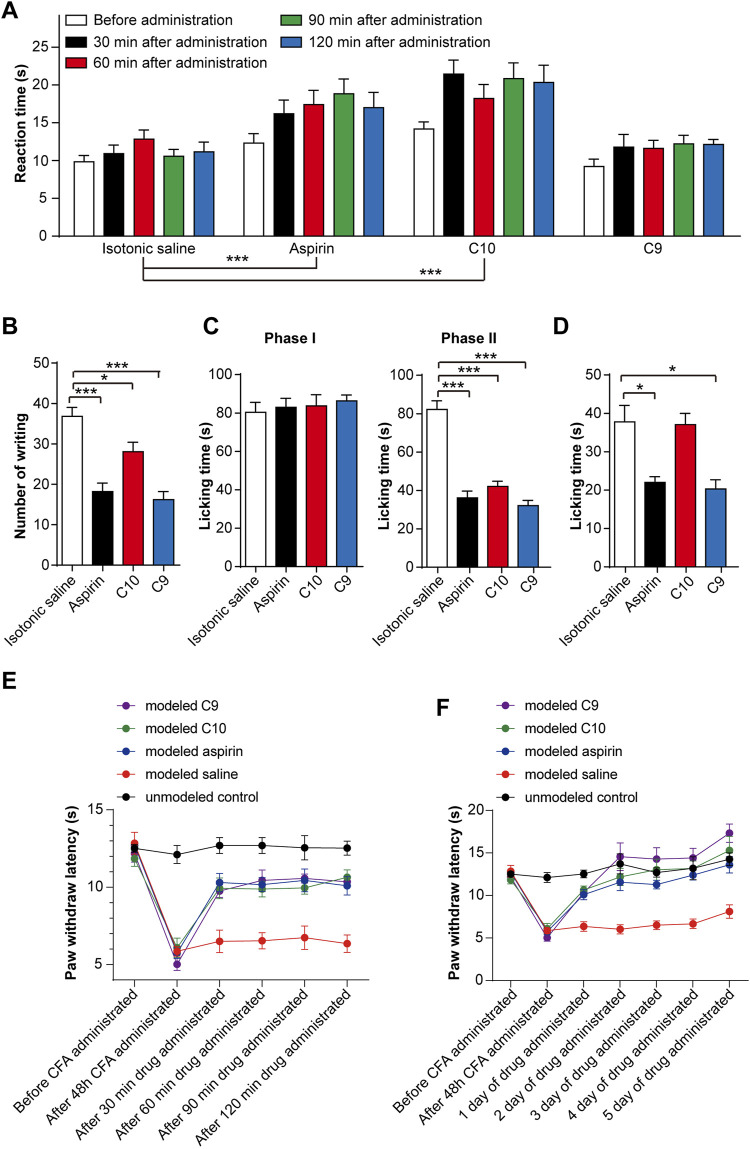
Effects of C10 and C9 on animal pain tests. **(A)** Effect of C10 and C9 against the hot plate test. Statistical assessments were performed by two-way ANOVA and Dunnett’s test comparing each group to the isotonic saline group; ***, *p* < 0.001. **(B)** Effect of C10 and C9 against the capsaicin test. **(C)** Effect of C10 and C9 against formalin-induced licking in the first phase and second phase in mice. **(D)** Effect of C10 and C9 against capsaicin-induced licking in mice. Statistical assessments **(B–D)** were performed by one-way ANOVA and Dunnett’s test comparing each group (*n* = 10) to the isotonic saline group; *, *p* < 0.05; ***, *p* < 0.001. **(E)** Short-term analgesic effect of C10 and C9 on CFA-induced inflammatory pain rats. Statistical assessments were performed by two-way ANOVA and Dunnett’s test comparing each group (*n* = 6) to the modeled saline group. The administration time had an impact on the administration effect (*p* < 0.0001). The statistical results indicated that the *p*-values between the modeled saline control group and the other groups were all less than 0.0001. **(F)** Long-term analgesic effect of C10 and C9 on CFA-induced inflammatory pain rats. Statistical assessments were performed by two-way ANOVA and Dunnett’s test comparing each group (*n* = 6) to the modeled saline group. The statistical results indicated that the *p*-values between the modeled saline group and the other groups were all less than 0.0001.

For observing the nociceptive activity of C10 and C9 on thermal-stimulated acute pain, the mice were injected intraperitoneally with isotonic saline, aspirin (20 mg/kg), C10 (20 µM), or C9 (4 µM), respectively. The pain threshold was tested at 30, 60, 90, and 120 min after administration. The application of aspirin for 60 min and 90 min, and C10 for 30 s, 90 s, and 120 min could alleviate acute pain caused by thermal stimulation in mice (Figure 3A). However, C9 had no effect on the acute pain caused by thermal stimulation. The phenotype of C10 is similar to that of aspirin (Chen S., et al., 2018), suggesting that C10 is an effective component of the TSS for resisting thermally-stimulated acute pain.

#### 3.2.2 Acetic acid-induced writhing test

To discover the nociceptive activity of C10 and C9 on chemically stimulated acute visceral pain, the mice were injected intraperitoneally with isotonic saline, aspirin (20 mg/kg), C10 (20 µM), or C9 (4 µM) separately. Sixty mins after administration, 0.4 mL of 1% acetic acid solution was intraperitoneally injected. The number of twists per mouse was recorded within 0–15 min. Compared with the control, aspirin, C10, and C9 could significantly inhibit the number of writhing events induced by acetic acid (Figure 3B), indicating that the pain threshold of the mice significantly increased. This is similar to TSS (Chen S., et al., 2018), indicating C10 and C9 as effective components of TSS for alleviating acute visceral pain caused by glacial acetic acid in mice.

#### 3.2.3 Formalin and capsaicin tests

For understanding the nociceptive activity of C10 on chemically stimulated acute pain, the mice were treated with isotonic saline, aspirin (20 mg/kg), C10 (20 µM), or C9 (4 µM). After 60 min of administration, 0.02 mL of 5% formalin solution was injected into the right hindfoot of the mice, and the cumulative licking time of each mouse in the first and second phases was recorded. Compared with the control, aspirin, C10, and C9 remarkably inhibited the licking time in the second phase, but did not alter the licking time in the first phase (Figure 3C), similar to the TSS phenotype (Chen S., et al., 2018). This suggests that both C10 and C9 are analgesic components of the TSS.

In addition, we tested the effects of C9 and C10 in the capsaicin-induced pain model. The mice were treated with isotonic saline, aspirin (20 mg/kg), C10 (20 µM), and C9 (4 µM), respectively. Aspirin and C9 decreased the time spent by mice licking their injected paws (Figure 3D). This indicated that the capsaicin-induced pain was significantly inhibited by aspirin and C9 but not by C10. These results showed that C9 specifically relieved capsaicin-induced acute neuropathic pain.

#### 3.2.4 CFA-induced chronic inflammatory pain model rats

The rats were divided into five groups as follows: unmodelled control, modeled saline, modeled aspirin, modeled C9, and modeled C10 groups. By comparing the thermal radiation pain thresholds of rats in the unmodelled control and the modeled groups, the CFA-induced chronic inflammatory pain model was successfully established after 48 h. After successful modeling, in order to determine the optimal administration time for analgesic effects, the thermal radiation pain response thresholds of the rats were measured on the first day of administration for 30, 60, 90, and 120 min. These results indicate that aspirin, C9, and C10 all exhibited analgesic effects with C9 showing the best analgesic effect 120 min after administration (Figure 3E). Therefore, during continuous administration, the thermal radiation pain threshold of the rats was 120 min after administration. After 5 consecutive days of administration, all data were statistically analyzed. After continuous administration for 5 days, both C9 and C10 exhibited long-term analgesic effects on chronic inflammatory pain similar to that of aspirin (Figure 3F).

Thus, both C10 and C9 can relieve pain, although their action mechanisms were different.

### 3.3 Characteristics and mechanism of C10 in enhancing mIPSC

#### 3.3.1 Time- and concentration-dependent effects of C10 on mIPSC

The abovementioned experimental results indicated that the absence of C9 and C10 both abolished the increased frequency of mIPSC (Figures 1A–C). This suggested that C10 and C9 may be potential active analgesic ingredients of TSS. To further confirm the effective components for increasing mIPSC frequency, we tested the effects of C9 with different concentrations on cultured cortical neurons. The results exhibited that the frequency of mIPSC was not increased by the application of the C9 component with concentrations of 0.2/2/20 μM (Supplementary Figures S1A, B). This result excluded the role of C9 in increasing the frequency of mIPSC. Subsequently, we investigated the role of C10 in cortical neurons.

**FIGURE 4:**
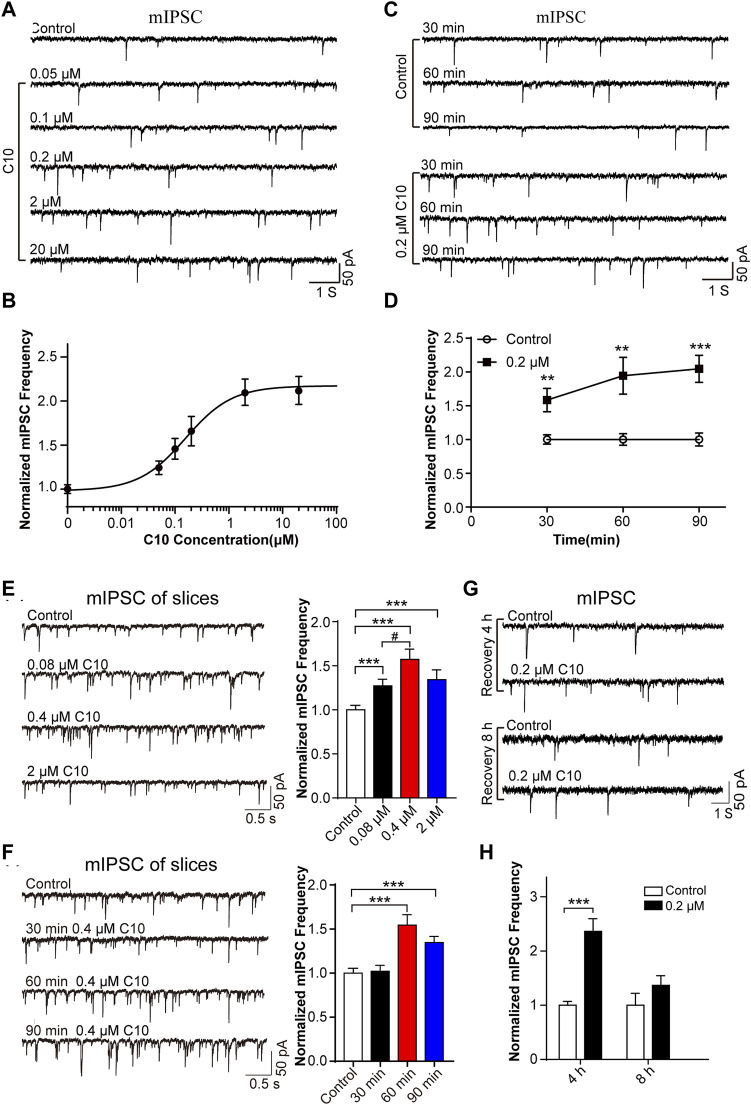
Time and concentration dependence of the effects of C10 on mIPSC. **(A, B)** The example traces and normalized mIPSC frequency recorded from cultured cortical neurons with different concentrations (0.05, 0.1, 0.2, 2, and 20 µM) of C10 treatment for 60 min, separately. The EC50 of C10 was 0.1563 ± 0.0272 µM analyzed by Hill fitting. **(C, D)** The example traces and normalized mIPSC frequency recorded from cultured cortical neurons with treatment of 0.2 μM C10 for 30, 60, and 90 min, individually. **(E)** The example traces (left) and normalized frequency (right) of mIPSC recorded from cortical neurons in brain slices with different concentrations (0.08 µM, 0.4 µM, and 2 µM) of C10 treatment for 60 min, individually. **(F)** The example traces (left) and normalized frequency (right) of mIPSCs recorded from cortical neurons in brain slices with 0.4 µM C10 for 30, 60, and 90 min, individually. **(G, H)** The example traces and normalized mIPSC frequency measured from cultured cortical neurons for 4 or 8 h restoring after 0.2 uM C10 treatment for 60 min. At least 25 cells from three independent cultures for **(B, D)**, 19 cells from six mice, and 15 cells from three independent cultures for **(H)** were analyzed. Statistical assessments **(D–F)** were performed by one-way ANOVA and Dunnett’s test comparing each group to the control group. Statistical assessments **(H)** were performed by *t*-test. **, *p* < 0.01; ***, *p* < 0.001.

Firstly, we analyzed the time- and concentration-dependent effects of C10 on mIPSC. We monitored the mIPSC of cultured cortical neurons after incubating cultured neurons with different concentrations of C10 (0.05, 0.1, 0.2, 2, and 20 µM) for 60 min, respectively. Within a certain range, as the concentration of C10 increased, the frequency of mIPSC gradually increased (Figures 4A, B). When C10 exceeded 1 μM, the frequency of mIPSC was unable to increase continuously. According to the Hill fitting, the EC 50 was 0.1563 ± 0.0272 µM. Then, we monitored the mIPSC of cultured cortical neurons after incubation with 0.2 µM C10 for 30, 60, and 90 min, respectively. Compared with the control, incubation with C10 for 30, 60, or 90 min could all increase the frequency of mIPSC (Figures 4C, D). As the incubation time was prolonged, the increased effect became more apparent.

Secondly, we also tested the concentrations and time dependence of C10 in brain slices. The slices were applied with different concentrations of C10 (0.08, 0.4, and 2 µM) for 60 min separately. According to the obtained mIPSC, all the C10 group (including 0.08/0.4/2 µM) had significantly increased frequency of mIPSC (Figure 4E), in accordance with the results of cultured neurons. We also tested the time dependence of C10 in neurons of the cortex slices (Figure 4F). Consistently, incubating slices with C10 for 60 or 90 min could both increase the frequency of mIPSC. However, incubating slices with C10 for 30 min did not change the frequency of mIPSC, which was inconsistent with the results in cultured neurons. Maybe a longer time is required for the drug to diffuse and work in the slices because of the large number of cells in the brain slice and tissue thickness. These results once again proved that C10 enhanced the inhibitory synaptic transduction of neurons in mouse cortex with a time- and concentration-dependent manner.

Thirdly, we investigated the enhancing duration of inhibitory synaptic transduction induced by C10 in mouse cortical neurons. C10 in 0.2 μM was applied in the cultured neurons for 60 min. The cells were then washed and incubated in a culture medium for 4 or 8 h for recovery. The recorded mIPSC revealed that, compared to neurons without C10 incubation, the frequency of mIPSC still significantly increased after 4 h of restoration (Figures 4G, H). However, after 8 h of restoration, the phenomenon of mIPSC frequency-increase disappeared (Figures 4G, H). This indicates that the action of C10 could last for at least 4 h.

In summary, the above experimental results unanimously support that C10 is an effective analgesic active component of TSS, and its role is related to enhancing inhibitory synaptic signaling.

#### 3.3.2 Effects of C10 on synaptic signaling transduction

To elucidate the role of C10 in synaptic signal transduction, we recorded the action potential evoked IPSC and mEPSC from cultured cortical neurons with an application of 0.2 or 2 μM C10. The frequency and amplitude of the mEPSC (Supplementary Figures S2A, B) and the amplitude of the evoked IPSC (Supplementary Figures S2C, D) remained unchanged with the C10 treatment. It seems that C10 only affects the inhibitory spontaneous synaptic signal transmission process rather than the induced rapid vesicle release or excitatory spontaneous release, which is consistent with the phenotype of TSS (Chen S., et al., 2018). These data further confirm that C10 is an active ingredient of TSS.

#### 3.3.3 C10 modulating mIPSC through the GABA signaling pathway related to the GABA_A_ receptor

To further elucidate the molecular mechanism of C10 analgesia, we predicted possible signaling pathways or targets of C10 based on its known molecular structure. Briefly, possible signaling pathways or potential targets were predicted by using the Swiss Target Prediction database, and then analyzed with the Kyoto Encyclopedia of Genes and Genomes (KEGG). The categories were arranged from top to bottom according to *p*-value (Supplementary Figure S2A). The results indicate that 21 possible signaling pathways may be potential targets for C10 active components, including the neuroactive ligand-receptor interaction, calcium signaling pathway, cGMP-PKG signaling pathway, and TRP channels. Also, a protein interaction network was generated to analyze the interaction relationships based on the predicted potential target genes (Supplementary Figure S3B).

Based on the above predictions and the results of previous studies, we selected three pathways that were more likely to be associated with C10 analgesia: the cAMP-PKA signaling pathway, TRPV1 receptor, and GABA receptor for further experimental verification.

Initially, the cAMP of neurons that were incubated for 60 min with or without 1 μM C10 was tested by ELISA. The presence of C10 did not influence cAMP content in neurons (Supplementary Figure S4A). Moreover, the mIPSC were recorded from the neurons with the addition of the PKA-pathway inhibitor H-89 (1 μM). The presence of H-89 did not alter the increase in mIPSC frequency when neurons were treated with C10 (Supplementary Figure S4B). Hence, the two experimental results indicate that the analgesic effect of C10 is not mediated through the cAMP–PKA signaling pathway.

Previous studies have shown that TSS inhibits TRPV1 channel currents in DRG neurons and produces analgesic effects (Chen et al., 2022). Thus, we investigated whether TRPV1 is a potential target of C10 by using TRPV1 agonists and antagonists. The mIPSC were recorded from brain slices under the condition of TRPV1 channel opening or closing, with or without 0.4 μM C10 incubation. Statistical analysis revealed that capsaicin increased the frequency of mIPSC to a certain extent when activating the TRPV1 receptor with or without C10 (Supplementary Figure S4C). When the TRPV1 receptor was closed by CPZ, the frequency of mIPSC recovered. After incubation with C10, the mIPSC frequency of the neurons increased independent of the state of the TPRV1 channel. Thus, the analgesic effect of C10 may not be related to TRPV1.

**FIGURE 5:**
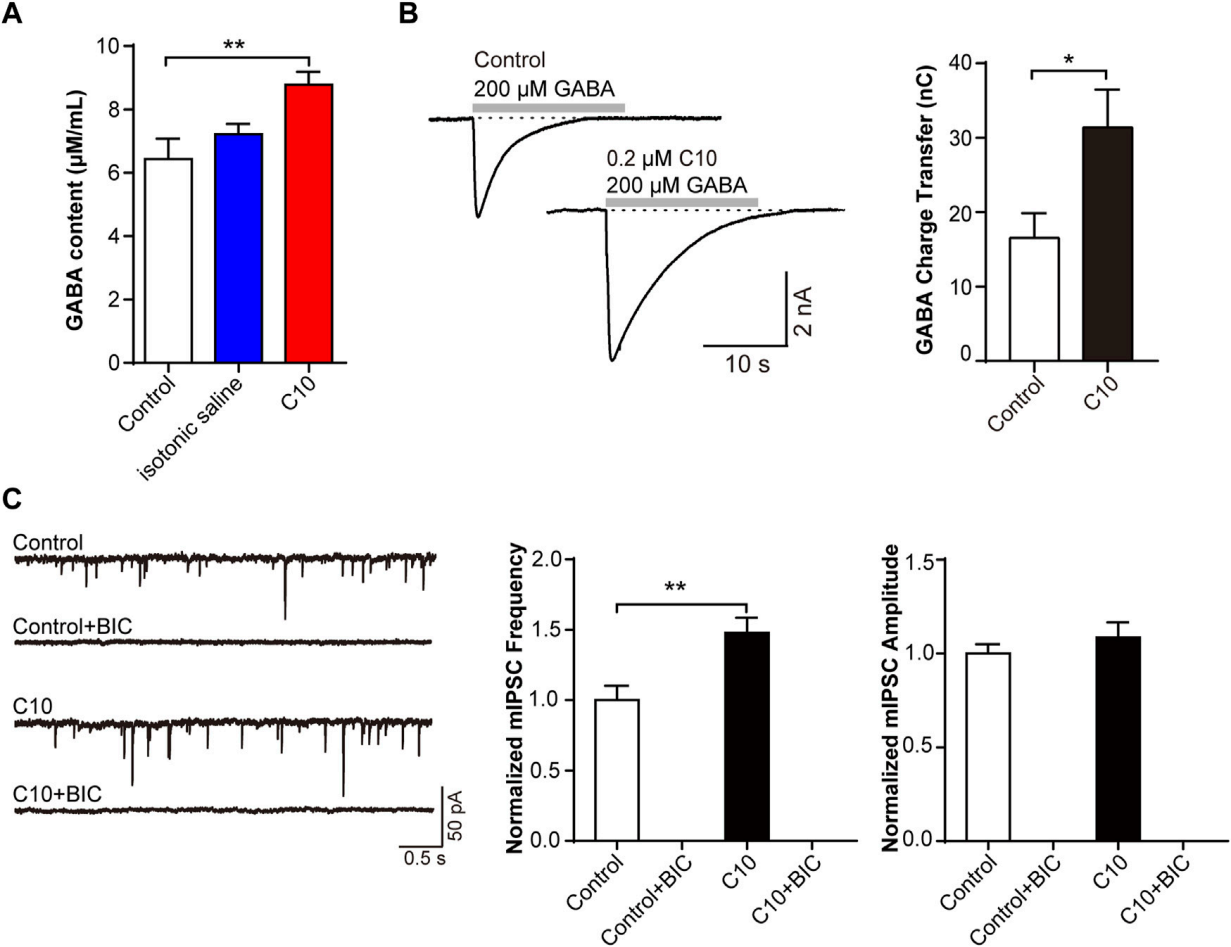
C10 modulating mIPSC through the GABA signaling pathway that related to GABA_A_ receptors **(A)** The GABA content of the brain cortex from the CFA animal models treated with or without C10 was quantified by ELISA assay. Control, the normal rat group; isotonic saline, the CFA model group; and C10, the CFA model group treated with 20 µM C10 with a content of 0 mL/10 g. The data of each group came from seven animals. **(B)** The example traces and charge transfer induced by 20-s 200 μM GABA recorded from cultured cortical neurons incubated with or without 0.2 µM C10 for 60 min. At least 9 cells from three independent cultures were analyzed. **(C)** The example traces, mIPSC frequency, and amplitude recorded from cultured cortical neurons incubated with or without 0.2 µM C10 for 60 min. BIC, bicuculline, the GABA_A_ receptor specific competitive antagonists. At least 18 cells from four independent cultures were analyzed. Statistical assessments **(A)** were performed by one-way ANOVA and Dunnett’s test comparing each group to the control group. Statistical assessments **(B, C)** were performed by *t*-test. *, *p* < 0.05; **, *p* < 0.01.

Next, we verified the involvement of the GABA receptor in the C10. The CFA pain model was prepared and treated with C10 for 5 days for analgesia. The GABA content of the rat cerebral cortex was measured using ELISA. C10 increased the content of GABA in the cerebral cortex of rats after C10 administration (Figure 5A). We then measured the GABA-induced charge transfer in neurons treated with or without C10. Interestingly, C10 exposure significantly increased the total charge transfer with 200 μmol/L GABA application (Figure 5B). This is consistent with the results of our previous study reporting that TSS increases GABA-induced charge transfer, further confirming Y-50 as an active ingredient in TSS analgesia.

MIPSC were monitored in brain slices with or without C10 incubation. The mIPSC frequency was always increased by C10. The specific competitive antagonist bicuculline (BIC) was used to inhibit GABA_A_ receptors. Statistical analysis exhibited that upon BIC application, the mIPSC signals were almost completely suppressed, even with the addition of C10 (Figure 5C). This indicated that almost all the mIPSC signals we recorded were mediated by GABA_A_ receptors, including the increase in the mIPSC signals induced by C10.

The above data clarified that C10-increased inhibitory synaptic signal transduction is mediated by the GABA signaling pathway, which is related to GABA_A_ receptors.

### 3.4 Characteristics and mechanism of C9 modulating TRPV1

#### 3.4.1 Concentration-dependent inhibition of C9 on CAP-induced TRPV1 currents

**FIGURE 6:**
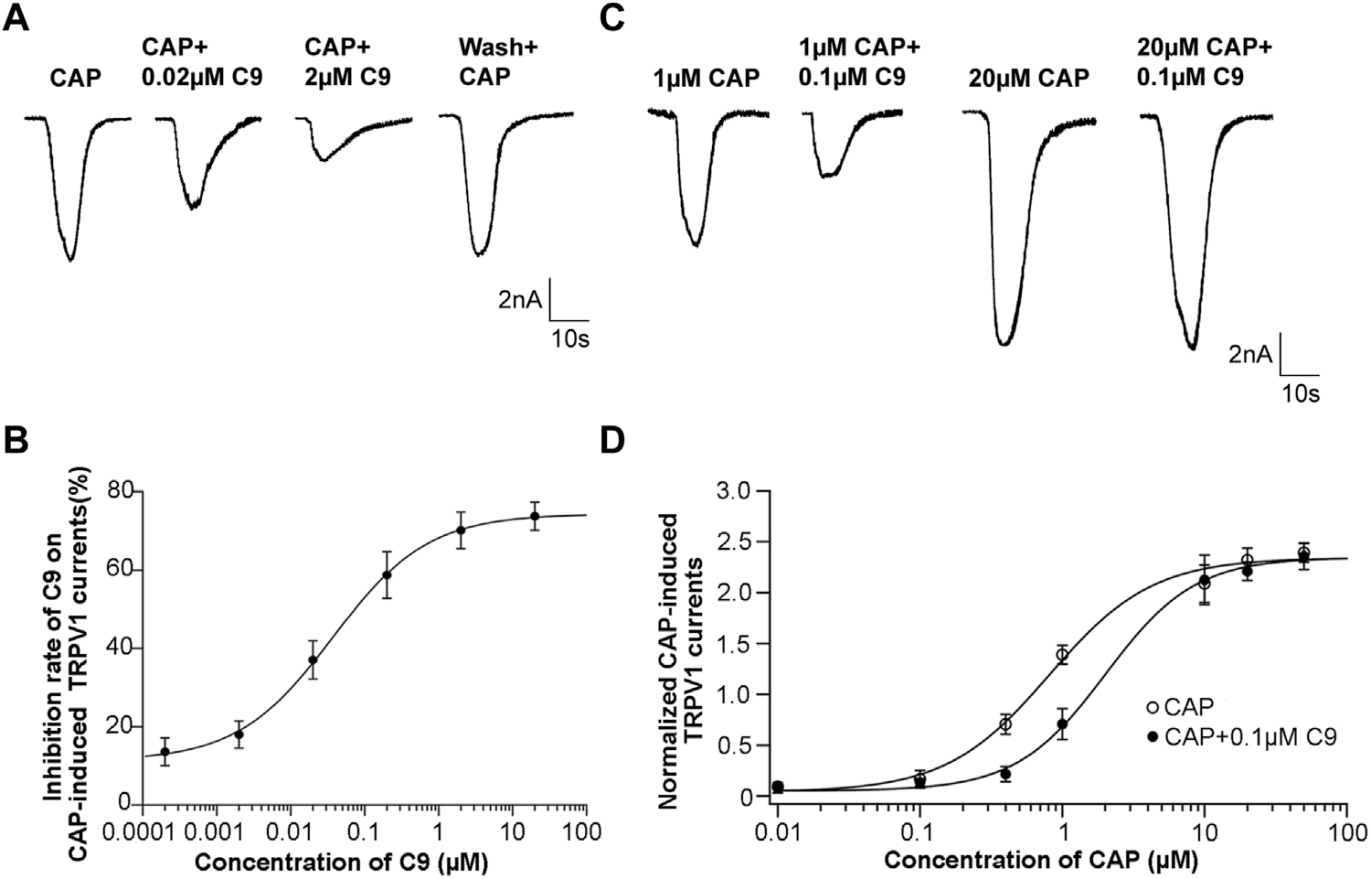
Concentration dependence and competitive antagonism of the effects of C9 on TRPV1 currents induced by CAP. **(A)** The example traces of TRPV1 currents recorded from DRG neurons with different concentrations (0.0002, 0.002, 0.02, 0.2, 2, and 20 µM) of C9 treatment, separately. **(B)** Concentration dependence curve of the inhibition on CAP-induced TRPV1 currents of C9. The EC50 of C9 was 0.0369 ± 0.0044 µM analyzed by Hill fitting. **(C)** Example trace of TRPV1 currents recorded from DRG neurons treated with 0.1 µM C9 in the application of different capsaicin concentrations (0.01 µM, 0.1 µM, 0.4 µM, 1 μM, 10 μM, 20 μM, and 50 µM). **(D)** Concentration dependent curves of TRPV1 currents induced by CAP in the presence or absence of C9. The number of DRG neurons in each group was no less than six.

To observe the effect of different C9 concentrations on the peak value of the CAP-induced TRPV1 current, the inhibition rate of the drug on the peak value of the CAP-induced TRPV1 current was calculated by multiplying the ratio of the difference between the peak current before and after administration and the peak current before administration by 100%. C9 inhibited the CAP-induced TRPV1 currents in a concentration dependent manner (Figure 6A). The inhibition rate of CAP-induced TRPV1 current by 2 μM of C9 could reach over 70%. Using the Hill equation to fit the concentration dependence curve of C9 inhibiting CAP-induced TRPV1 current, we could obtain the half effective concentration as (0.0369 ± 0.0044) μM and the Hill coefficient of (0.6748 ± 0.0540) (Figure 6B).

#### 3.4.2 C9 competitively antagonize TRPV1

DRG neurons were treated with different concentrations of CAP, and the concentration dependence of the peak value of the CAP-induced TRPV1 current was observed. A concentration-dependent curve was drawn using the concentration of CAP as the abscissa and the normalized TRPV1 current value as the ordinate. The normalized TRPV1 current value is calculated by dividing the recorded peak value of CAP-induced TRPV1 current by the average value of the peak value of 0.5 μM CAP-induced TRPV1 current. Subsequently, the mixture of CAP with different concentrations and 0.1 μM C9 was given to DRG neurons, and then we also observed whether the concentration dependence of the peak value of CAP-induced TRPV1 current changed in the presence of 0.1 μM C9 (Figure 6C).

Accordingly, concentration-dependence curves were plotted. The two concentration-dependent curves were compared. If the peak value of the high concentration of CAP-induced TRPV1 current did not decrease in the presence of 0.1 μM C9, it indicated that C9 competed with CAP to antagonize TRPV1. By contrast, the peak value of the high concentration CAP-induced TRPV1 current decreased, indicating that C9 and CAP non-competitively antagonized TRPV1. As presented in Figures 6C, D, when 0.1 μM C9 existed, the normalized TRPV1 current value induced by 50 uM CAP was 2.346 ± 0.545, and no significant difference (*p* > 0.05) was observed when 0.1 μM C9 was not obtained (Figure 6D). The above results showed that there is a competitive antagonistic relationship between C9 and CAP when TRPV1 is inhibited.

#### 3.4.3 Effects of C9 on TRPV1 expression

**FIGURE 7:**
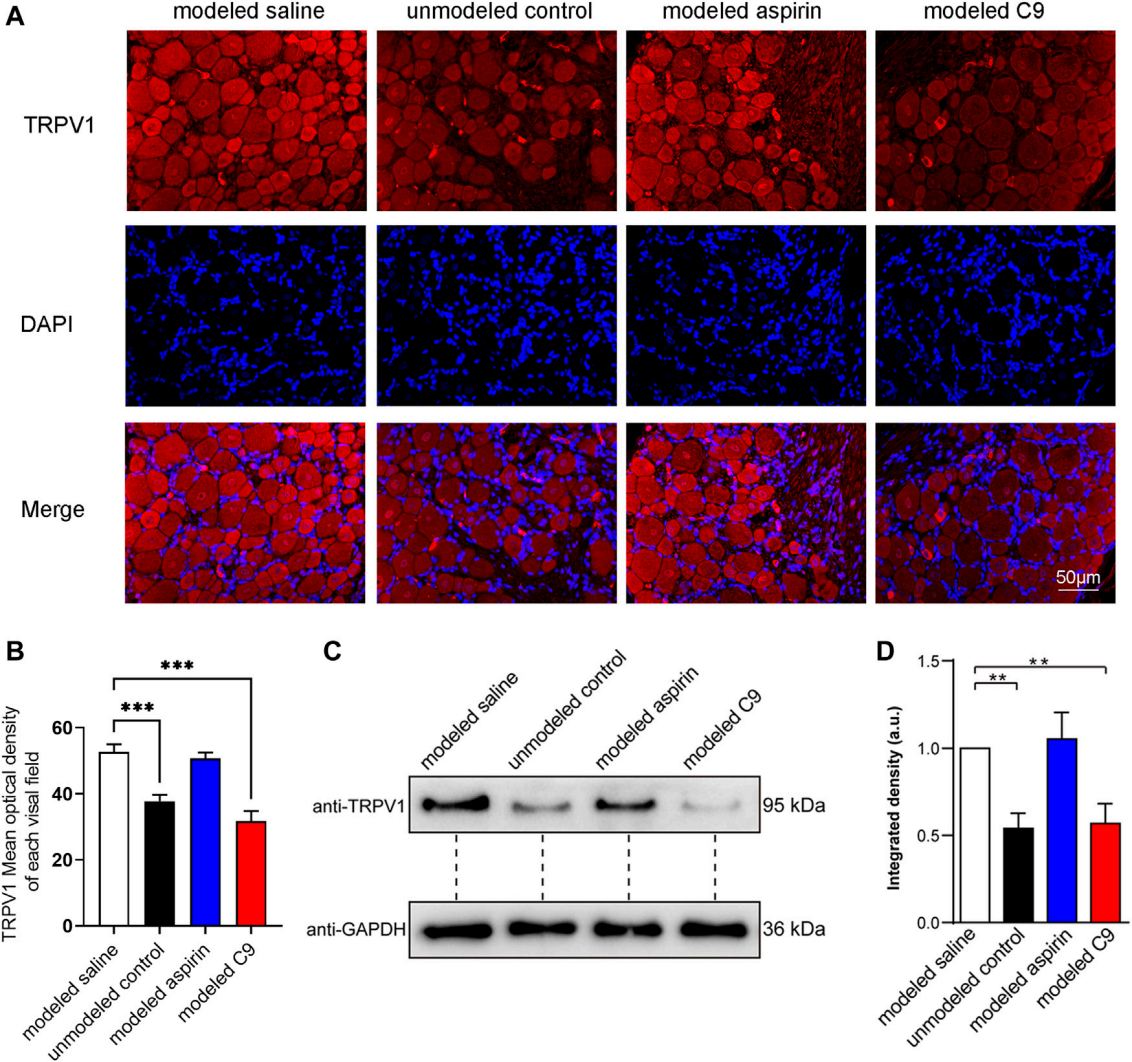
Effects of C9 on TRPV1 expression by using immunofluorescence and WB assay. **(A)** Immunofluorescence showed the expression of TRPV1 receptor protein in DRG neurons. Scale bar, 50 μm. **(B)** The fluorescence optical intensity of TRPV1 protein. The number of slices read per group was not less than five. **(C)** An example of WB experiment results also showed the expression of TRPV1 receptor protein in DRG neurons. **(D)** The standardization ratio of TRPV1 to GAPDH band densities in DRG neurons of various drug-treated CFA-induced inflammation pain rats (Four batches for all the groups). Statistical analysis was performed using one-way ANOVA and Dunnett’s test comparing each group to the modeled saline group. **, *p* < 0.01; ***, *p* < 0.001.

After the experiment on chronic inflammatory pain induced by CFA in rats, DRG was used for immunofluorescence and Western blotting (WB) assays to observe the expression level of TRPV1. The results of immunofluorescence results were consistent with those of WB assay (Figure 7).

The TRPV1 protein expression in the modeled saline group was significantly increased compared with that in the unmodeled control group. This indicates that chronic inflammatory pain could upregulate the TRPV1 expression in rat DRG neurons. The expression of TRPV1 protein in rat DRG neurons did not change in the modeled aspirin group compared with that in the modeled saline group, indicating that aspirin did not regulate TRPV1 protein expression, and its analgesic effect was not related to TRPV1. The expression of TRPV1 protein in DRG neurons of rats in the modeled C9 group was significantly downregulated compared with that in the modeled saline group. Thus, C9 can downregulate the expression of TRPV1 protein, which is closely related to the analgesic effect of C9 in rats with chronic inflammatory pain.

## 4 Discussion

Our previous studies have demonstrated that the analgesic effect of TSS was achieved through two mechanisms, one is increasing the frequency of mIPSC in mouse cerebral cortex neurons (Chen S., et al., 2018) and the other is modulating TRPV1 in DRG neurons (Chen et al., 2022). However, as TSS is a multi-component mixture, which component or components in TSS produce analgesic effects through the aforementioned mechanisms is unclear. In this study, by screening the components from triterpenoid saponins, we identified that component C10 and component C9 increased the frequency of mIPSC in mouse cortical neurons and modulated the TRPV1 in DRG neurons, respectively. Both C10 and C9 effectively relieved pain in various animal models (Figure 2). These results indicated that the analgesic effect of TSS was mainly produced by its two components, C10 and C9, through different analgesic pathways.

Based on the previously reported results (Chen et al., 2014), we obtained a total of ten types of components from TSS. We studied compounds 6 and 7 in the early stages and did not observe good analgesic effects; therefore, these two components were excluded from this screening. Given that the amount of eight components extracted and separated was not large, to screen the key active ingredient from the eight components in a small amount, we did not use the method of screening by separately observing the modulatory effect of a certain component on mIPSC and TPRV1. Instead, the eight components were initially mixed to detect their modulatory effects on mIPSC and TRPV1.

After determining that the component mixture could increase the frequency of mIPSC in mouse cortical neurons and modulate TRPV1 in DRG neurons, we removed one component from the mixture each time and observed the modulatory effect of the remaining mixture composed of seven components on mIPSC and TPRV1. Subsequently, the effect of removing one component was compared with that of the original mixture of eight components. The component with the most significant difference in effect that was removed from the mixture is the key active ingredient. Using this method, we quickly and in small amounts screened out the key active ingredients, C9 and C10, that regulate mIPSC and TRPV1, respectively.

The relationship between inhibitory synaptic transmission and pain relief has been fully recognized (Vanghan and Christie, 1997; Funai et al., 2014; Leonardon et al., 2022). In our previous studies, we have found that TSS alleviated pain by increasing the frequency of mIPSC (Chen S., et al., 2018), but the specific analgesic mechanism remains unclear. Here, we showed that the phenotype of the component C10 is very similar to that of TSS, not only increasing the frequency of mIPSC in cortical neurons (Figures 1, 4) but also exhibiting good analgesic effects in animal pain models (Chen S., et al., 2018). Our results indicates that C10 is the main component of TSS in the cerebral cortex for pain relief.

To further identify the analgesic targets of C10, we utilized network pharmacology to predict potential signaling pathways based on the structure of C10. C10 increased the frequency of mIPSC and was associated with GABA_A_ receptors. Our data indicated that C10 not only increased the release of GABA, but also enhanced the GABA_A_ receptor-mediated charge transfer induced by GABA (Figures 5A, B).

γ-Aminobutyric acid subtype receptors, a kind of pentameric ligand-gated chloride ion channel receptor, are the most prominent mediators of inhibitory signals in the central nervous system and play a pivotal role in anesthesia (Zhou and Guan, 2021). GABA_A_ receptors are heteropentamers, consisting of subunits from six α subunits (α1-6), three *ß* subunits (β1-3), three *γ* subunits (γ1-3), and others (Zhu et al., 2018). Although the amino acid sequence, expression level, and localization of each subunit in brain tissue species have been well studied, how many different isomers are formed by their interactions and how these isomers function, in cooperation or separately, remain unclear (Ghit et al., 2021; Arias et al., 2023). The most common GABA_A_Rs in the brain are composed of two α1, two β2, and one γ2 arranged γ2β2α1β2α1 counterclockwise (as shown in the abstract graph) (Daniel and Ohman, 2009; Ghit et al., 2021). The role of GABA_A_ receptors in inhibitory currents is influenced by subunit composition and arrangement (Minier and Sigel, 2004; Hannan et al., 2020). The mature receptor has a central chloride ion channel that is gated by the neurotransmitter GABA and is regulated by many general anesthetics, such as propofol and etomidate (Zhou and Guan, 2021; Ghit et al., 2021; Chen Q., et al., 2018; Alvarez and Pecci, 2018). GABA_A_ receptors are mainly distributed in the postsynaptic of synapse, where GABA neurotransmitters can bind to GABA_A_ receptors, causing chloride ion channels to open and increasing intracellular anions in milliseconds, leading to cell membrane depolarization, thus mediating the transmission of inhibitory signals. GABA_A_ receptors are widely reported to be involved in the regulation of pain signals and chronic pain status. These anesthetics, which target GABA_A_ receptors used in clinical settings, although effective, also have certain side effects. Therefore, new analgesics that targeting GABA_A_ receptor need to be developed.

Interestingly, we identified that C10, a traditional Chinese medicinal ingredient derived from natural plants, has an analgesic effect on the GABA_A_ receptors. In our data, we found that the component C10 from TSS could increase inhibitory neural signal transduction (Figures 1, 4). The release of GABA in neurons and GABA-induced currents both increased after being treated with C10 (Figures 5A, B). However, the inhibitory effects of GABA in the cortex are mediated by two types of receptors: ionic GABA_A_ receptors and metabotropic GABA_B_ receptors. The GABA-induced rapid current was theoretically mediated by GABA_A_ receptors (Figure 5B) and almost all the mIPSC signals were inhibited by the specific inhibitor BIC of GABA_A_ receptors (Figure 5C). Thus, it can be reasonably inferred that C10 increases inhibitory synaptic signal transduction by modulating the GABA signaling pathway, which is mediated by GABA_A_ receptors.

In our experiment, the neurons were treated with C10 for 60 min. Hence, we may not completely rule out the role of GABA_B_ here. C10 enhanced inhibitory synaptic signal transduction in cortical neurons by increasing the GABA release. Whether C10 acts directly or indirectly on the GABA_A_ receptors remains unclear. According to the literature, GABA_A_R agonists play important roles in anesthesia and sedation (Zhou and Guan, 2021). Etomidate, propofol, and barbiturates directly activate GABA_A_Rs (Garcia et al., 2010). Propofol is a dialkyl phenol that directly binds to the α+/β− or α+/γ− interface of GABA_A_R (Maldifassi et al., 2016). Barbiturates, a kind of commonly used anesthetic drug, bind to TMD in α+/β− interface of the GABA_A_R (Chiara et al., 2016). Benzodiazepines, of which the core chemical structure is the fusion of benzene ring and diazo heterocycle, bind to the N-terminal at binding sites α/β and α/γ interfaces, and increase Cl^−^ conductance (Sigel and Lüscher, 2011). Etomidate, an imidazole type intravenous hypnotic, binding to the β+/α− interface of the GABA_A_R, enhance the activity of GABA_A_R to quickly induce general anesthesia (Chiara et al., 2012; Stewart, et al., 2013). However, we compared the structure of C10 with these known drugs that can directly bind to GABA_A_R and observed that the structural similarity is low. In addition, we used molecular docking to predict the interaction between C10 and GABA_A_R and identified a very small probability of direct interaction. Therefore, C10 may indirectly regulate the GABA_A_R receptor-mediated signaling pathway. Indeed, more research is needed to elucidate these regulatory mechanisms.

TRPV1 is highly enriched in DRG and its involvement in inflammatory pain is well documented (Wu et al., 2021). During inflammation, many signaling pathways and inflammatory mediators can cause aberrant peripheral neuronal activity (Sondermann et al., 2019). Importantly, many of these pathways converge and sensitize TRPV1 (Salazar et al., 2009). TRPV1 reacts to a variety of both chemical and physical stimuli (Caterina et al., 1997). In most cases, these stimuli cause the opening of a pore in the channel-receptor complex and elicit a transmembrane ion current. In particular, the data show that extracellular acidification of the environment increases TRPV1 receptors’ sensitivity to capsaicin (Ryu et al., 2003). Since acidification of the environment is an important sign of a developing inflammatory response (Grivennikov et al., 2010), potentiation of TRPV1 receptors, when combined with the effect of other agonists (e.g., CAP), can be considered part of the signaling mechanism triggered in a cell in response to inflammation (Tsvetkov et al., 2017).

In a model of acute inflammatory pain, C9 demonstrated similar or even better analgesic effects than aspirin in terms of body-twisting response stimulated by glacial acetic acid or in terms of the paw-licking response stimulated by formalin and capsaicin. The results of patch-clamp experiments indicated that the peak value of TRPV1 current activated by CAP recorded on DRG neurons could be rapidly reduced by the transient addition of C9. Further research revealed that the relationship between C9 and CAP was competitive antagonism, that is to say C9 was similar to CPZ and was also a competitive antagonist of TRPV1.

Sa Xiao et al. have reported that all allosteric regulatory ligands significantly inhibit capsaicin-induced Ca^2+^ influx through the TRPV1 channel (Xiao et al., 2019). The molecular structure analysis showed that CPZ, AMG517, loureirin B, and tetrahydropalmatine all have similar H receptor/donor parts and substituted benzene ring moiety, while the hydrophobic aliphatic side chain portion was significantly different. From the structure of C9, it could be seen that its aglycone was hederagenin. Hederagenin also has abundant H receptor/donor parts and a substituted benzene ring moiety, whereas the hydrophobic aliphatic side chain portion is greatly different. From the results of component screening, the different structure is also important. Because the aglycone of C10 is also hederagenin, C10 did not induce a significant regulatory effect on TRPV1, similar to C9. Among the eight screened components, only the aglycones of C9 and C10 were hederagenin. This also shows their unique structure in terms of pain relief. In fact, there have been literature reports that hederaginin itself could produce analgesic effects by regulating TRPV1 (Zhang, et al., 2020).

In addition to blocking TRPV1 activation, reducing the expression or TRPV1 via siRNA transfection, pharmacological intervention, and other methods can dramatically alleviate inflammatory and neuropathic pain (Warwick et al., 2019). Zheyin Wang et al. have reported that baicalin ameliorates neuropathic pain by suppressing TRPV1 upregulation in the DRG of rats with neuropathic pain (Wang Z., et al., 2020). Moreover, Ping Li et al. found that baicalin might play a promising analgesic role in the DRG of rats implanted with tumor cells by preventing the upregulation of TRPV1 (Li et al., 2020). He-Ya Qian et al. reported an effective analgesic effect of metformin on mechanical allodynia in rats with bone cancer pain, which may be mediated by the downregulation of ASIC3 and TRPV1 (Qian et al., 2021).

The results of WB and immunofluorescence experiments revealed that after 5 days of successful modeling, the expression of TRPV1 was upregulated in DRG neurons of CFA-induced chronic inflammatory pain rats. Continuous administration of C9 for 5 days effectively restored the upregulated TRPV1 to normal levels. These results indicated that the pharmacological intervention with C9 downregulates TRPV1 and alleviates inflammatory pain.

It is worth mentioning that, initially, we believed that both C9 and C10 could produce good analgesic effects and that there may be some overlap in their action mechanisms. However, the experimental results indicate that the receptors and pathways regulated by the two compounds are completely different. Although the aglycones of both compounds are hederagenin, the significant difference in the action target may be attributed to the differences in the groups connected to the hederagenins. However, it cannot be concluded that the roles of TRPV1 and postsynaptic response in pain generation and transmission are not completely without intersections. A study by Daisuke Uta et al. provides fundamental evidence that chronic inflammation and neuropathic pain models amplify the release of glutamate by activating TRPV1 in central axon terminals (Uta et al., 2019).

Further, we will investigate the analgesic effects of C9 and C10 in different combinations, which may yield unexpected results. Nevertheless, TSS produces analgesic effects through different components C9 and C10, through peripheral and central analgesic pathways, respectively, which precisely demonstrates the characteristics of the multi-component, multi-target, and multi-pathway effects of traditional Chinese medicine and provides a lateral pharmacological basis for “a traditional Chinese medicine is a small compound".

## 5 Conclusion

Our results revealed that C10 and C9 are effective analgesic components in TSS. Their analgesic mechanism is to act on neurons in the cerebral cortex and DRG neurons in the spinal cord, respectively. C10 exhibited analgesic effect by increasing inhibitory synaptic signal transduction related to GABA_A_ receptors in the cerebral cortical neurons, while C9 showed analgesic effect through modulating TRPV1 in DRG neurons.

## Data Availability

The original contributions presented in the study are included in the article/Supplementary Material, further inquiries can be directed to the corresponding authors.

## Abbreviations

TSS, triterpenoid saponins extracted from Stauntonia chinensis; TRPV1, transient receptor potential vanilloid l; SD, Sprague–Dawley; mIPSC, miniature inhibitory postsynaptic current; DRG, dorsal root ganglion; IPSCs, inhibitory postsynaptic currents; CNQX, 6-cyano-7-nitroquinoxaline-2,3-dione; mEPSC, miniature excitatory postsynaptic current; PTX, picrotoxin; TTX, tetrodotoxin; CAP, capsaicin; CPZ, capsazepine; MOD, mean optical density; SEM, standard error of the mean; ANOVA, analysis of variance; BIC, bicuculline.
