# Supplementary material for: Central and peripheral analgesic active components of triterpenoid saponins from Stauntonia chinensis and their action mechanism
Source: Front Pharmacol. 2023 Oct 16;14:1275041. doi: 10.3389/fphar.2023.1275041 (PMC10613692; doi:10.3389/fphar.2023.1275041)
Supplement: Supplementary file 1 [file Table1.DOCX]

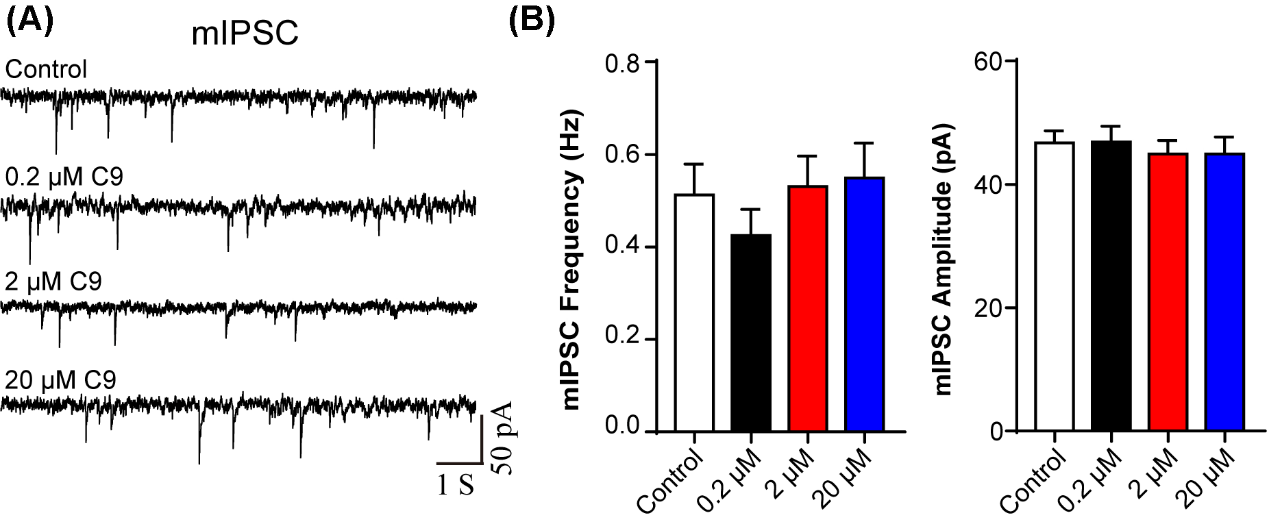


**Figure S1 C9 has no effects on increasing mIPSCs.**

(**a**) Example traces of mIPSC were recorded from the cultured cortical neurons treated with C9 (0.2/2/20 µM) for 60 minutes, independently. (**b**) Statistical summary of frequency and amplitude of the mIPSC described in (a). Data information: shown are means±SEM. At least 24 cells from 6 independent cultures were analyzed in each group. Statistical assessments were performed by One-Way ANOVA.


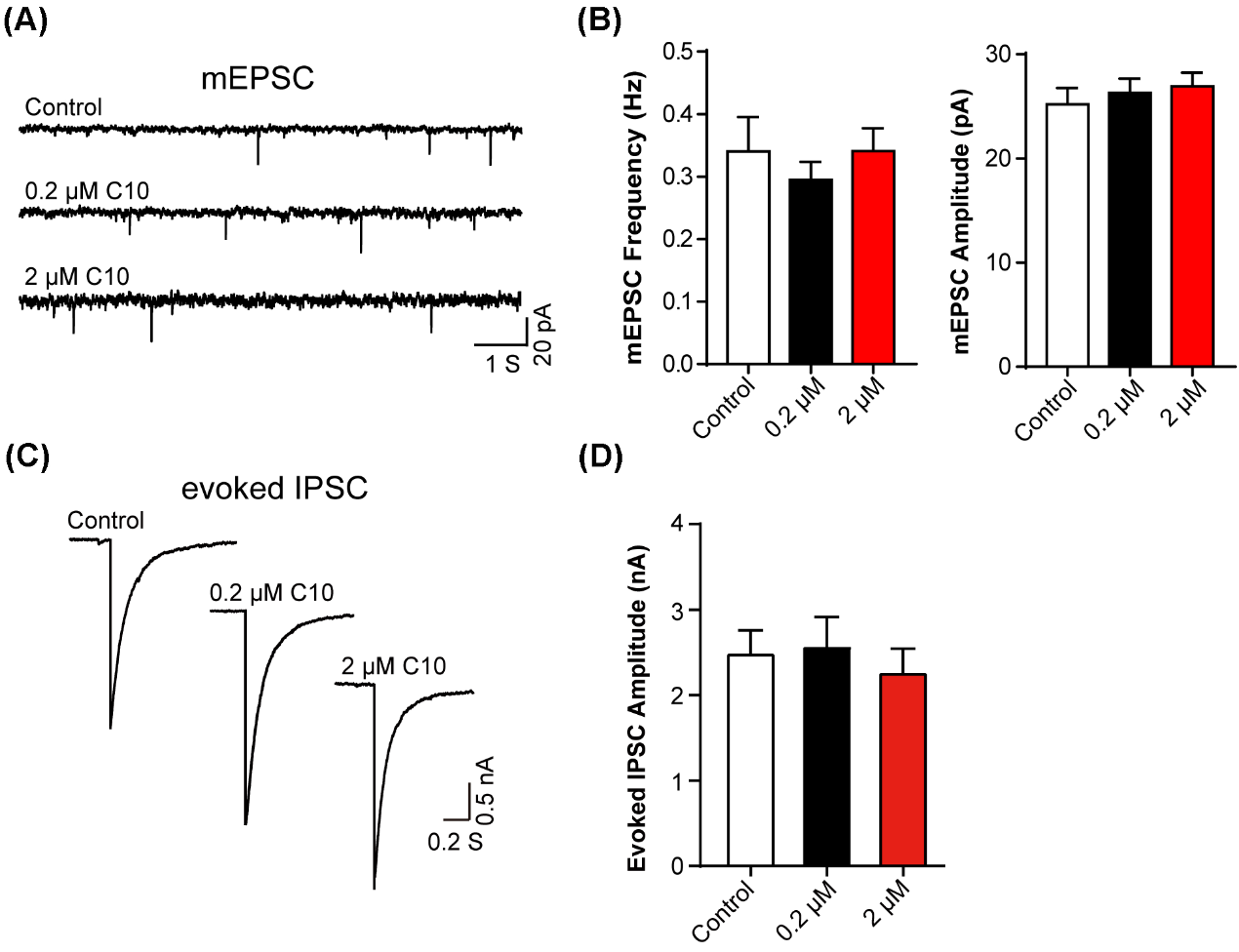


**Figure S2 C10 did not alter excitatory synaptic signaling transduction and evoked IPSC.**

(**a**) Example traces of mEPSC were recorded from the cultured cortical neurons incubated with C10 (0.2/2 µM) for 60 minutes. (**b**) Statistical summary of frequency and amplitude of the mEPSC described in (a). (**c**) Represent traces of evoked IPSC measured from cultured cortical neurons treated with 0.2 or 2 μM C10. (**d**) Summary graph of amplitude of the evoked IPSCs described in (c). Data information: shown are means±SEM. At least 14 cells of 3 cultures were analyzed. Statistical assessments were performed by One-Way ANOVA with multiple comparisons comparing each condition to control.


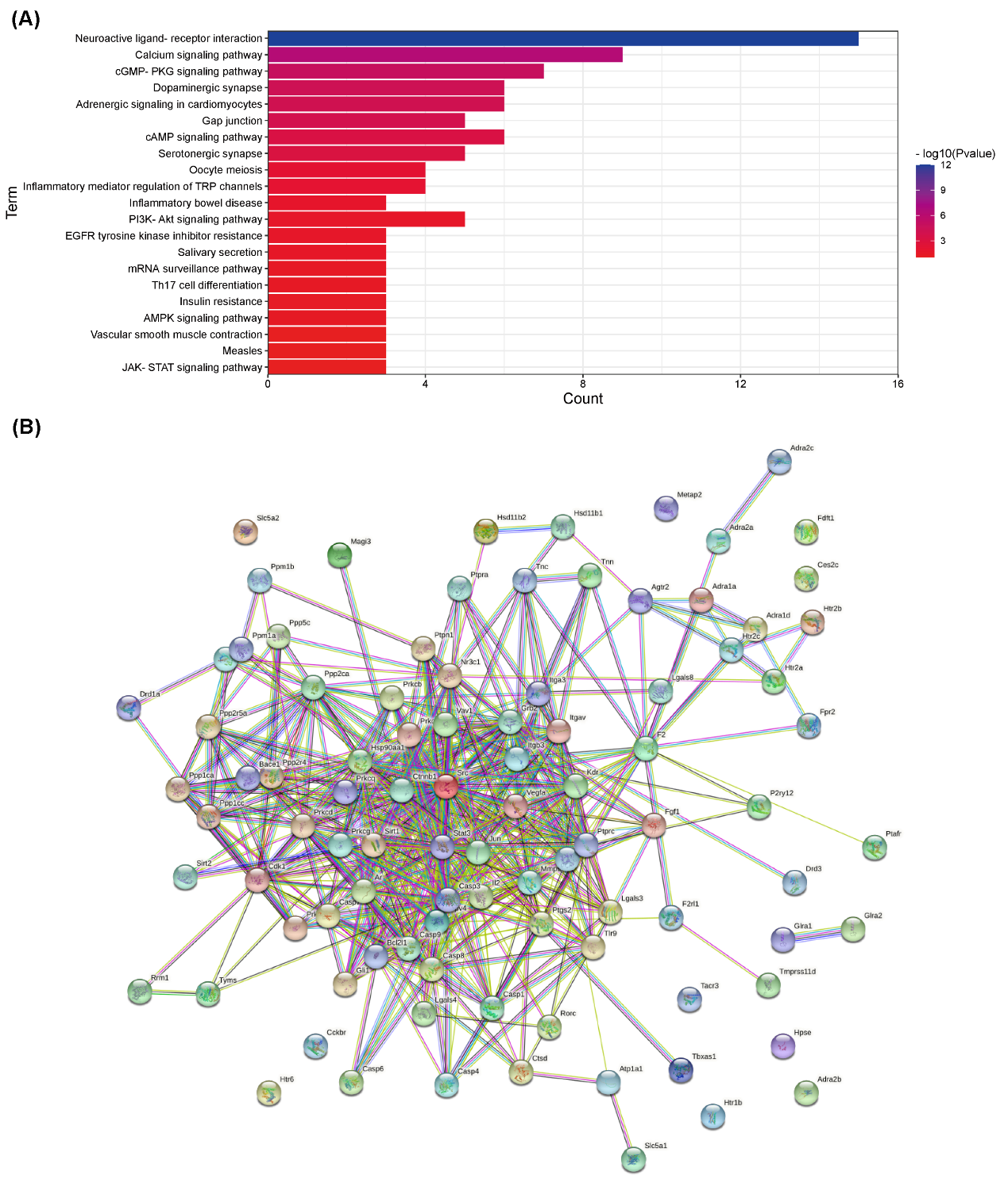


**Figure S3 The KEGG analysis and protein interaction network of C10 potential targets.**

(**a**) The KEGG analysis of the possible signaling pathways or targets of C10 according to the known molecular structure. Shown are the categories arranged from top to bottom with P-value size. (**b**) The protein interaction network was predicted by using potential target genes.


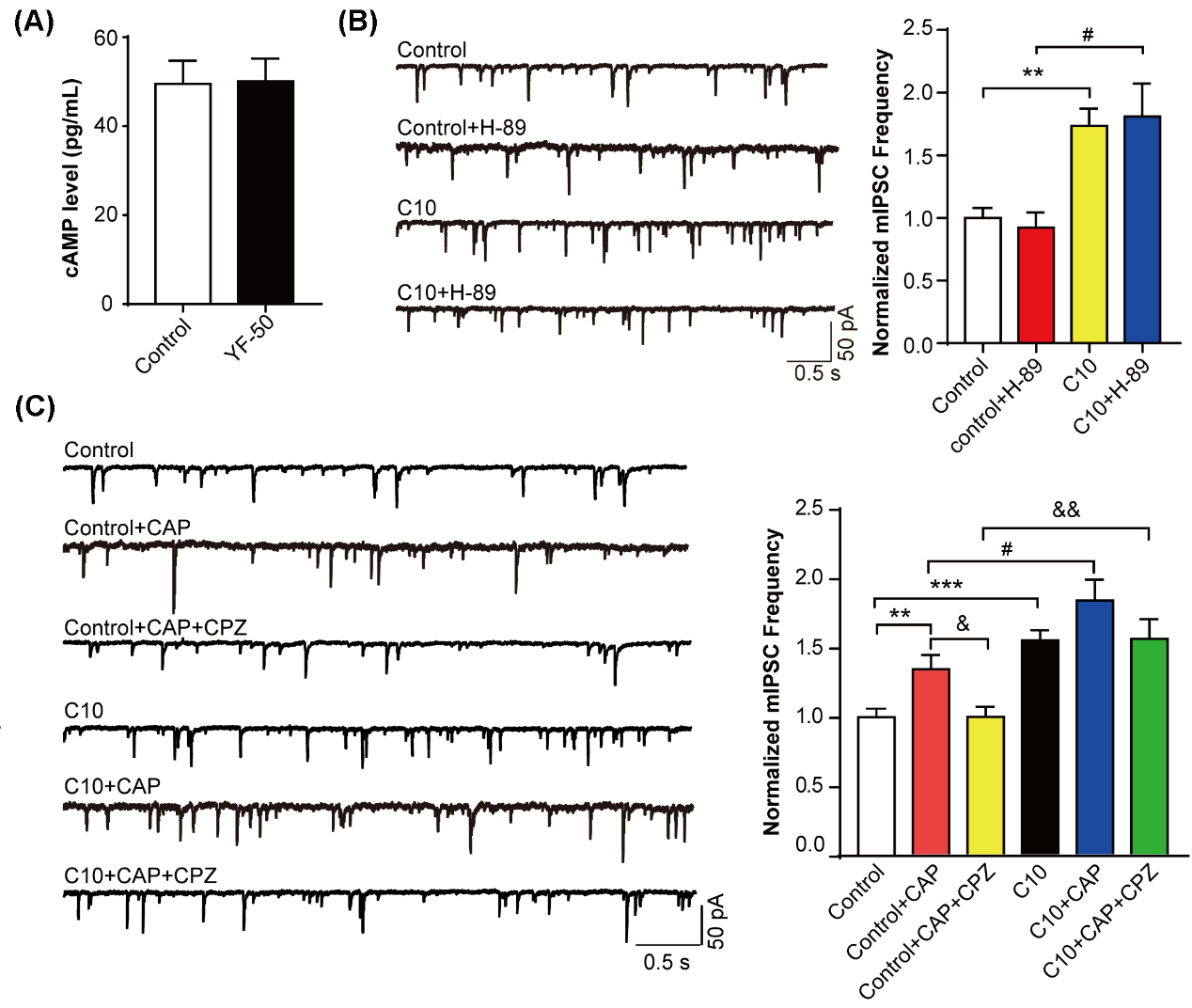


**Figure S4 The function of C10 is unconcerned with cAMP-PKA signaling pathway and TRPV1 channel.**

(**a**) The cAMP level measured from neurons treated with or without 1 µM C10 for 60 minutes. (**b**) Example traces of mIPSC were recorded from the neurons with the addition of PKA-pathway inhibitor H-89 (1 μM) after incubation of 1 μM C10. (**c**) The example traces and normalized frequency of mIPSC recorded from cortical neurons in brain slices with the addition of TRPV1 receptor specific agonists (CAP, 10 μM) or/and antagonist (CPZ, 10 μM) after cells incubation with or without 0.4 μM C10 for 60 minutes. Data information: shown are means±SEM. In (b), *n*=9 neurons were analyzed from three mice. In (**c**), *n*≥16 neurons from 4 mice werre analyzed. Statistical assessments were performed by One-Way ANOVA with multiple comparisons. **, *P* < 0.01; ***, *P*< 0.001; #, *P* < 0.05; &, *P* < 0.05; &&, *P* < 0.01.
